# A Disease-Mediated Trophic Cascade in the Serengeti and its Implications for Ecosystem C

**DOI:** 10.1371/journal.pbio.1000210

**Published:** 2009-09-29

**Authors:** Ricardo M. Holdo, Anthony R. E. Sinclair, Andrew P. Dobson, Kristine L. Metzger, Benjamin M. Bolker, Mark E. Ritchie, Robert D. Holt

**Affiliations:** 1 Department of Biology, University of Florida, Gainesville, Florida, United States of America; 2 Department of Zoology, University of British Columbia, Vancouver, British Columbia, Canada; 3 Department of Ecology and Evolutionary Biology, Princeton University, Princeton, New Jersey, United States of America; 4 Department of Biology, Syracuse University, Syracuse, New York, United States of America; Imperial College, United Kingdom

## Abstract

Tree cover is a fundamental structural characteristic and driver of ecosystem processes in terrestrial ecosystems, and trees are a major global carbon (C) sink. Fire and herbivores have been hypothesized to play dominant roles in regulating trees in African savannas, but the evidence for this is conflicting. Moving up a trophic scale, the factors that regulate fire occurrence and herbivores, such as disease and predation, are poorly understood for any given ecosystem. We used a Bayesian state-space model to show that the wildebeest population irruption that followed disease (rinderpest) eradication in the Serengeti ecosystem of East Africa led to a widespread reduction in the extent of fire and an ongoing recovery of the tree population. This supports the hypothesis that disease has played a key role in the regulation of this ecosystem. We then link our state-space model with theoretical and empirical results quantifying the effects of grazing and fire on soil carbon to predict that this cascade may have led to important shifts in the size of pools of C stored in soil and biomass. Our results suggest that the dynamics of herbivores and fire are tightly coupled at landscape scales, that fire exerts clear top-down effects on tree density, and that disease outbreaks in dominant herbivores can lead to complex trophic cascades in savanna ecosystems. We propose that the long-term status of the Serengeti and other intensely grazed savannas as sources or sinks for C may be fundamentally linked to the control of disease outbreaks and poaching.

## Abstract 2

The removal of rinderpest had cascading effects on herbivore populations, fire, tree density, and even ecosystem carbon in the Serengeti ecosystem of East Africa.

## Author Summary

Diseases are known to play important roles in regulating and structuring populations, but the consequences of disease outbreaks for entire communities and ecosystems are not as well understood. The Serengeti wildebeest were historically kept at low numbers by the rinderpest virus, but underwent a population explosion (irruption) after rinderpest was eradicated in the 1960s. We examined nearly a half-century of data to test the hypothesis that this irruption was responsible for a decline in the frequency of fires in this ecosystem (through increased grazing and a reduction in fuel loads), and that this in turn increased the density of trees. We found strong evidence for this indirect link between rinderpest and tree density, and less support for the role of other factors such as elephants and climate. We also investigated the consequences of this chain of events for ecosystem carbon, and suggest that the combined effects of increased grazing intensity by wildebeest, reduced fire, and increasing tree density may have shifted the Serengeti from being a net source to a net sink for carbon. This would imply that seemingly small ecological perturbations such as disease outbreaks have the potential to profoundly affect ecosystem function.

## Introduction

In addition to being a prominent structural feature of savanna and forest ecosystems, tree cover has far-reaching consequences for ecosystem function [1],[2]. Trees are a key component of stored carbon (C), and thus important in the potential for ecosystems to act as carbon dioxide (CO_2_) sinks in the effort to curb global warming. Despite this, understanding the factors that influence tree cover, herbaceous production, and soil organic matter in savannas and other nonforest biomes remains a vexing and challenging problem in ecology [3],[4]. It has been hypothesized that top-down limitation by fire and herbivores plays a dominant role in regulating tree cover within bounds determined by rainfall [5]. Although rainfall does indeed appear to impose an upper limit on tree cover in savanna ecosystems [5]–[7], evidence to support the role of fire and herbivores as factors driving tree cover below this maximum is conflicting [4]–[6],[8]. There has accordingly long been disagreement among ecologists about the relative importance of climate, fire, and herbivores (especially elephants) as determinants of tree-to-grass ratios and tree cover in African savannas [3],[9],[10]. Studies at the next trophic level do little to clarify the situation as the factors that regulate herbivores (such as disease and predation) and fire occurrence are poorly understood for any given ecosystem.

**Figure 1:**
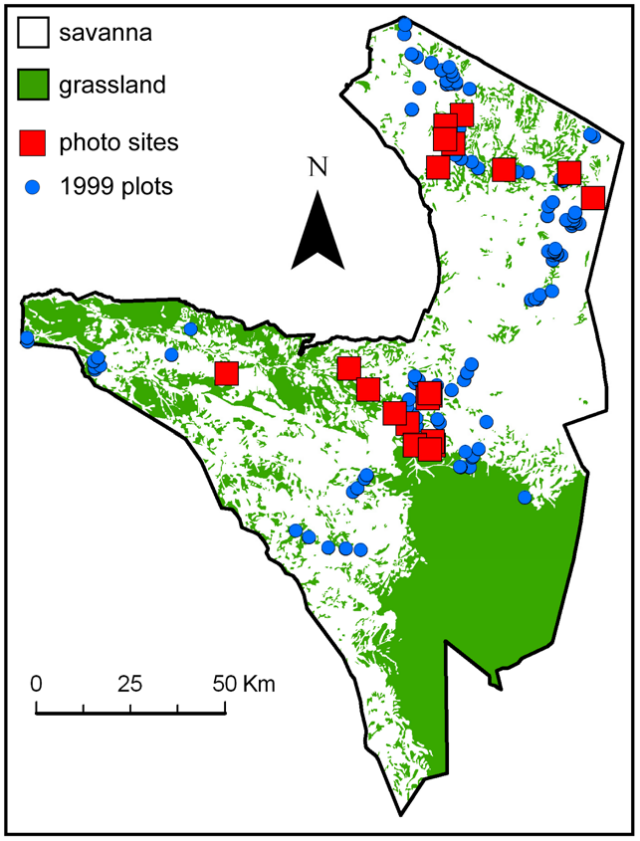
Map of Serengeti National Park with tree density sampling sites. Shown are locations of photopanorama sites and sampling sites for the 1999 tree density data (only those in savanna sites are shown) [40]. The map also illustrates the main savanna and grassland habitat types. It appears to show fewer than 51 photopanorama sites because several of these were taken close together but with different cardinal orientations.

We drew on a 44-y time series (1960–2003) to identify the direct and indirect links among disease, herbivores, fire, rainfall, and changes in tree density (which we use here as a measure of tree cover) in the 25,000 km^2^ Serengeti-Mara ecosystem of East Africa (Figure 1). Elephants (the dominant browsers), fire, and wildebeest (the dominant grazers) have all been proposed as important drivers contributing to changes in tree cover [11]–[14]. It has been suggested that rinderpest eradication set in motion a far-reaching and ongoing regulatory trophic cascade throughout the ecosystem, with the resulting irruption of wildebeest leading to a reduction of grass biomass and fire frequency, and an increase in tree cover [15]–[17]. Here we use a rigorous statistical approach to examine the evidence for this cascade, as well as competing explanations for historic patterns of fire prevalence and fluctuations in tree density. We further examine how changes at various nodes in this cascade (herbivores, fire, and trees) may have shifted the carbon (C) balance of the Serengeti ecosystem over the past half-century.

We compared ten competing models for the determinants of fire and tree density change in this ecosystem (Table 1). These models jointly investigated the effects of grazer abundance and rainfall on fire, and the influence of fire, elephants, grazers, rainfall, and atmospheric CO_2_ concentration on per capita changes in tree density inferred from photopanoramas.

**Table 1:** Candidate models of fire and per capita tree density change in the Serengeti.

Model^a^	Variables Affecting:		p_D_ b	DIC^c^
	Fire	Trees		
1	*R*_w∶d_	*F*	50.9	362.6
2	*W*	*F*	49.3	355.7
**3**	***W*** **, ** ***R*** **_w∶d_**	***F***	**49.3**	**349.9**
4	*W*	*E*, *F*	52.6	359.0
5	*W*	*E*, *F*, *R*_ann_	52.6	358.2
6	*W*	*E*, *F*, *W*	51.7	357.8
7	*W*	*E*	51.9	358.0
8	*W*, *R*_w∶d_	*E*, *F*	51.3	351.7
9	*W*, *R*_w∶d_, *R*_ann_	*E*, *F*	52.0	352.7
10	*W*, *R*_w∶d_	*F*, *CO^2^*	51.7	351.1

a The models are defined by the variables that drive fire (*F*) and tree (*T*) dynamics: elephants (*E*), wildebeest (*W*), annual rainfall (*R*_ann_), wet∶dry season rainfall (*R*_w∶d_), and atmospheric CO^2^ concentration (*CO^2^*).

b Effective number of parameters.

c DIC; the best-fitting overall model (lowest DIC) is shown in bold.

## Results

**Figure 2:**
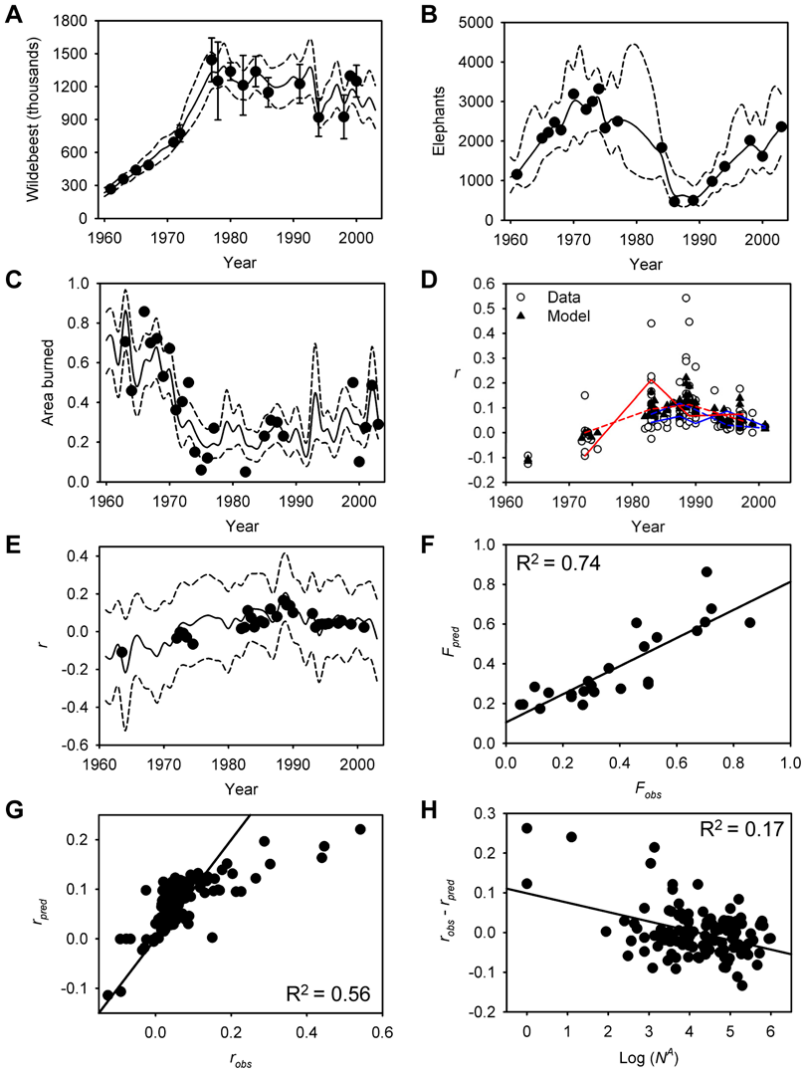
Fits to the data for the best model. (A) wildebeest, (B) elephants, and (C) proportion of Serengeti National Park burned. Each subfigure illustrates observations (filled circle), posterior means of estimated true values (solid line), and 95% credible intervals for the posterior distributions (dashed lines); standard errors for the observed values are shown for the wildebeest data. (D) Annualized rates of per capita tree density change (*r*), centred on the midpoints of each time span (e.g., a value of *r* based on photos taken in 1980 and 1990 is centred on 1985). Correlations among points (corresponding to photo sites) are not shown for legibility, except for two sites: (blue and red solid lines, data; dashed lines, model fit). (E) Model fit for rate of per capita tree density change (mean and 95% credible intervals) plotted jointly over time with observed values of *r* (the values are means for the midpoint values in [D]). Predicted versus observed values of (F) fire (proportion of area burned) and (G) rates of per capita tree density change, and (H) model residuals from (G) versus the logarithm of initial tree density corresponding to the start of each photo sequence.

The model with the strongest support, based on the deviance information criterion (DIC) (Table 1), identified wildebeest (Figure 2A, presumably via their grazing impact on grass biomass) and intra-annual variation in rainfall (the ratio of wet∶dry rainfall) as the best predictors of fire occurrence (defined as the proportion of the ecosystem that burns per year). The differences in model DIC values (Table 1) suggested that wildebeest grazing is a better predictor of fire than is intra-annual rainfall variation, but both of these variables contributed to the observed global patterns of fire occurrence in the Serengeti (Figure 2C) as inferred from the credible intervals of their coefficients (β_1_ and β_2_, respectively; Equation 4 and Table 2). The inclusion of mean annual rainfall did not improve model fit (Table 1).

**Table 2:** Estimated values for Bayesian state-space model parameters.

Parameter	Mean	SD	2.5%^a^	Median	97.5%^a^
*E*_0_	1,029	139	769	1,040	1,242
*W*_0_	236	22	203	232	286
*T*_0_	601	212	233	594	970
*a*	3.56	1.08	1.83	3.46	6.03
α	9.24	1.26	8.05	9.13	10.82
β_0_	0.07	0.64	−1.12	0.04	1.33
β_1_	−0.0019	0.0005	−0.0028	−0.0019	−0.0010
β_2_	0.22	0.09	0.04	0.22	0.39
γ_0_	0.19	0.07	0.06	0.19	0.35
γ_1_	0.46	0.19	0.11	0.45	0.88
*h*	909	528	77	861	2,142
*r*_E_	0.071	0.040	0.013	0.065	0.170
*r*_W_	0.194	0.033	0.127	0.194	0.259
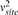	0.037	0.010	0.019	0.037	0.057
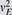	0.158	0.097	0.028	0.141	0.376
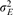	0.246	0.078	0.106	0.245	0.403
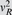	0.074	0.006	0.063	0.074	0.087
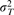	0.103	0.042	0.044	0.095	0.208
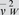	0.091	0.012	0.070	0.091	0.114
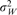	0.054	0.022	0.022	0.050	0.108

a 95% credible intervals.

SD, standard deviation.

The results also suggested that that fire alone—and not elephants (Figure 2B), mean annual rainfall, or atmospheric CO_2_—has been the primary driver of observed changes in tree density (Figure 2A–2E). Per capita tree density changes were negative from 1960 until the mid 1970s, becoming positive thereafter (decelerating after 1990); our model closely tracked these trends (Fig. 2D, 2E, and 2G). Furthermore, about a third of the variance in tree density change that was unexplained by the best-fitting model could be explained by variation in density (Figure 2H): photopanorama sequences with low initial tree density had faster per capita growth than expected, suggesting that density dependence (which we could not model explicitly, as we only had data on relative density changes within photopanorama sites) has also played an important role in regulating tree dynamics.

**Figure 3:**
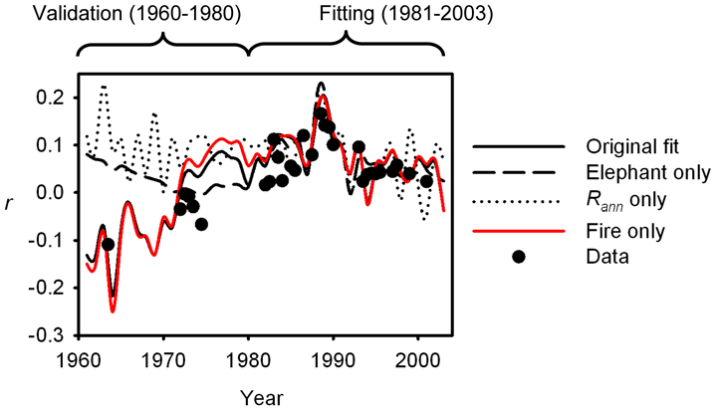
Validation of the role of fire with a restricted dataset. Models that incorporated only the effects of fire, elephants, or rainfall on per capita tree density change were fitted to photopanorama data from the post-1980 period only (1980–2003 data were used for model fitting). The figure shows how well alternative tree dynamics models fit the pre-1980 photopanorama data (the validation period). The original best-fit model (model 3, fitted to the entire dataset) is also plotted for reference. Each data point represents a mean across multiple sites for a particular time period (a midpoint, see Figure 1E).

The DIC results were clear in teasing apart the drivers of fire occurrence over time, but less clear in terms of inferring the factors regulating tree density. On the one hand, model 3 performed better than (or as well as) more complex models, but on the other, models 2 (fire effects only) and 7 (elephant effects only) had similar DIC values (Table 1). The role of fire was supported, however, by an examination of coefficient credible intervals. The fire coefficient (γ_1_) differed from zero both when it appeared alone or with elephants as a covariate (Table 1; values for model 3 given in Table 2), but the credible intervals for the elephant, mean annual rainfall, and atmospheric CO_2_ coefficients included zero in all models. To further test the explanatory power of fire versus other factors in driving tree density changes, we ran model 3 again, but fitted only to tree data for the period 1981–2003 (see Methods), and then validated by comparing its predictions with the reserved 1960–1980 data. We also ran two competing single-factor models (elephants and mean annual rainfall) with the same dataset. In all cases we included wildebeest and intra-annual rainfall variation as explanatory variables for fire. The fire model performed equally well with the reduced and full datasets (Figure 3), closely tracking the trajectory of the original model and predicting the decline in tree density that occurred in the 1960s and 70s (Figure 3). The other two models, however, while fitting the 1981–2003 data quite well, performed poorly for the validation period (Figure 3).

**Figure 4:**
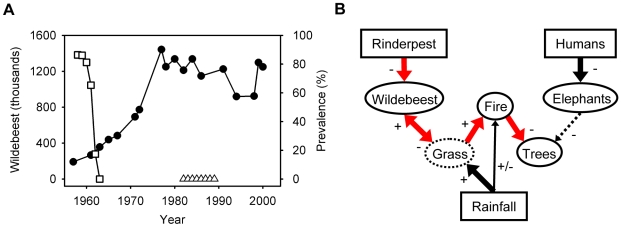
Rinderpest-mediated regulation of ecosystem dynamics. (A) Serengeti wildebeest population (filled circle) and rinderpest seroprevalence reported for the periods 1958–1963. (B) Inferred causal relationships driving tree population dynamics in the Serengeti. The dominant effects are shown with thick arrows. Highlighted in red is a four-step pathway of causality linking rinderpest with tree population dynamics. The grass compartment, as an unobserved variable, is shown in dotted outline.

We extended our analysis to include the role that the eradication of rinderpest (a *Morbillivirus* closely related to measles and distemper [16]) played in causing a shift from top-down disease control to bottom-up resource limitation in wildebeest. The prevalence of rinderpest, which causes high levels of mortality in wildebeest calves, declined rapidly following vaccination of the cattle that were a reservoir for the pathogen (Figure 4A) [16]. Eradication of the pathogen permitted the wildebeest population to erupt, ultimately driving the trophic cascade (driven by grazing-mediated fire suppression) that resulted in a marked increase in tree density.

**Figure 5:**
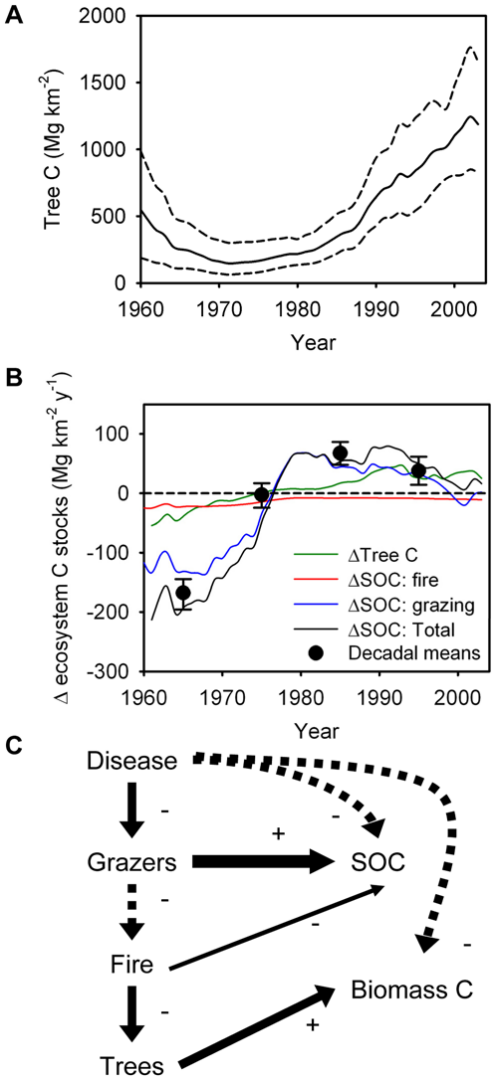
Shifts in ecosystem C balance. (A) Tree C was modelled with a point estimate of tree biomass C from 1999 [40]. Shown are the posterior mean (solid line) and 95% credible intervals (dashed lines). (B) Simulated changes (as 5-y moving averages) in ecosystem C stocks (total, tree C, and SOC changes driven by fire and grazing to 40 cm depth) and annualized decadal net changes in total ecosystem C balance (means±95% confidence intervals) between 1960–2003; the temporary shift from net sink to source predicted by our simulation in 2000 was driven by drought and resulting overgrazing. (C) Inferred causal pathways linking disease with changes in ecosystem C stocks as a result of a trophic cascade (solid line, direct effects; dashed line, indirect effects).

The rinderpest-triggered trophic cascade may have had far-reaching functional consequences for the role of savanna ecosystems as carbon (C) sources or sinks. The soil C (SOC) and plant biomass C pools contain most of the C in terrestrial ecosystems, and a decline in the size of these pools would make the ecosystem a net source of C. Grazing intensity (GI) and fire have been shown theoretically [18] and empirically (unpublished data) [19] to enhance and reduce the size of the soil organic matter pool in the Serengeti, respectively. We redefined tree density in units of C per km^−2^, and used functions relating fire and GI to changes in SOC to simulate changes in the size of these two C pools with a modified version of our best-fit Bayesian state-space model (BSS) model (model 3). The model predicted changes in ecosystem-level C stocks in the Serengeti between 1960 and 2003 based on annual estimates of GI, fire extent, and changes in tree density over this period (Figure 5).

## Discussion

Our results suggest that long time series, examined over appropriate spatial scales, can identify strong signals in the relationships among herbivores, fire, climate, and vegetation. Our model explained about three-quarters of the variance in both fire and per capita tree density change (Figure 2F–2H). This is particularly striking in the case of fire, which depends not only on fuel loads, but also on the occurrence of ignition events. Here we show that grazer population size (and by implication grazer-determined fuel loads) is a key determinant of fire frequency, a finding documented in at least one other savanna system [20], and thus grazer abundance is an important indirect driver of tree population dynamics, supporting findings from previous modelling and empirical studies [21]–[23]. Although much of the relationship between wildebeest population size and fire extent is arguably driven by the widespread changes that occurred up to 1975 in the immediate aftermath of rinderpest eradication, to the best of our knowledge no other plausible driver of fire extent has exhibited a temporal pattern that might explain the historic decline in fire. For example, marked increases in human population density around the Serengeti [24] and changes in park fire management policies over the past few decades (both of which alter the frequency of ignition events) [11] might have been expected to overwhelm the effects of grazers in determining fire occurrence, but this was clearly not the case. An important caveat to our model results is the lack of direct data on grass biomass across the ecosystem. The link between wildebeest population size and standing grass biomass is implicit in our model, and would no doubt be strengthened by the availability of time-series data for grass biomass. Other studies, however, have shown both directly [25] and indirectly (by estimating grass production and wildebeest consumption [21]) that wildebeest can exert a very strong regulatory effect on grass cover in the Serengeti at landscape scales. This finding is consistent with the observation that at large enough spatial scales, it is fuel loads rather than ignition events that determine fire occurrence in savannas [26]. Our results also support the hypothesis that savannas are primarily regulated by fire (and not rainfall) above a mean annual rainfall threshold of 650–700 mm (most of the Serengeti woodlands fall above this limit) [5],[22]. Variation in rainfall failed to directly explain patterns of tree density change, but it did play an indirect role by modulating the fire regime [27].

Notably, our results suggest that although elephants are known to exert important local effects on tree dynamics in Serengeti woodlands [12],[13],[28], there is only weak support for the notion that elephants have influenced ecosystem-wide temporal patterns in tree density over the past half-century. Our model suggests that fire, rather than elephants, has been the key driver of tree density change in the Serengeti over the past half-century. A separate simulation model, drawing on different sources of data, predicted that both fire and elephants (at their present-day population size, which is relatively high by historical standards) can determine tree cover in the Serengeti, with fire being of greater importance [21]. There are, however, additional factors that must be considered in evaluating the overall importance of elephants for tree density. First, the elephant population of the Serengeti has historically been kept low by poaching. It is rapidly expanding at present, and in the future elephants could potentially exert large-scale impacts on vegetation. Second, elephants are patchily distributed in Serengeti [21],[29], and global assessments as summarized in our model do not capture spatial heterogeneity in their effects (the same observation applies to fire), or localized interactions with fire and other factors [30],[31]. An important future challenge will be to reconstruct and explain spatial patterns of tree cover change in this system. Third, elephants may have impacts on tree cover in savannas that are not reflected by changes in tree density. This is because they often feed on medium to large trees [12], and their impact can reduce canopy cover (thus having an impact on vegetation structure) while maintaining density (or even potentially increasing it, as a single large tree is replaced by several smaller recruits).

Our results are consistent with the rinderpest trophic cascade hypothesis [15],[16], which proposes a linear chain of causality of remarkable simplicity operating in the Serengeti, one that zigzags vertically across three “trophic” levels: decreased pathogen→increased specialist consumer (wildebeest)→decreased producer (grass)→decreased generalist “consumer” (fire)→increased producer (trees), mediating the relative dominance of two functional producer groups, trees and grasses (Figure 4B). On the face of it, that a pathogen could regulate such a fundamentally important aspect of ecosystem structure as woody cover (through its effects on an herbivore that does not even consume trees) might seem improbable, but there is growing evidence of trophic cascades via subtle links in other ecosystems [32],[33], and, more broadly, increasing recognition of the role of pathogens in regulating plant communities [34]. We propose that the dominant factors controlling tree density in the Serengeti are top down, and that episodic top-down regulation of the herbivores by infectious disease has historically played an important role in restructuring this and (potentially) other ecosystems. In essence, the period of rinderpest enzoosis that prevailed throughout the first half of the 20th century in the Serengeti matches the scenario of the HSS (Hairston, Smith, and Slobodkin) “Green World” model [35], but with a pathogen playing the role of predator and fire dynamics modulated by herbivory constituting a critical piece of the puzzle [16]. Although the scheme we propose in Figure 4B simplifies the range of possible interactions and feedbacks that could occur in an ecosystem as complex as the Serengeti (e.g., food availability as mediated by rainfall could affect the susceptibility of herbivores to disease), it captures what we believe to be some of the salient features of the system.

Our simulations of C stocks suggest that the changes in wildebeest population density, fire prevalence, and tree density that have occurred over the past half-century may have had important effects on the C stocks in woody biomass (Figure 5A). Furthermore, new field studies show that current densities of wildebeest and resident grazers stimulate storage of soil C (unpublished data). Thus, our analysis allows us to estimate C loss and accumulation in the Serengeti ecosystem as a function of its trophic organization. A caveat to our estimates of tree biomass C is that, lacking data on changes in the size class distribution of tree over time, we must assume for simplicity that C stocks are directly proportional to density. This assumption might hold true when the size distribution is stable over time, but when changes in density are asymmetric across size classes (e.g., fire tends to remove small trees, elephants large ones), this assumption is violated. Better estimates of historic changes in tree C stocks will require data on tree size distribution changes over time, which are not yet available. Nevertheless, given our data, our best estimate is that Serengeti trees and soils constitute a net C sink, removing on the order of 40–70 Mg C km^−2^ y^−1^ from the atmosphere (Figure 5B). Across 25,000 km^2^ of mostly protected woodland habitat across the entire ecosystem, this is equivalent to 10^6^ Mg C y^−1^. In contrast, our model suggests that in the past, when rinderpest was endemic and grazer densities were low, the Serengeti was a net C source. Rinderpest eradication may thus have had ecological consequences in the Serengeti that extend beyond the impact on habitat and landscape structure in this system. Furthermore, any future epizootic (or any population crash from whatever cause, including disease, hunting, or drought) may rapidly reverse the changes that have occurred over the past few decades and would release the C from its present stored form back into the atmosphere.

A fundamental insight that emerges from the Serengeti longitudinal dataset is the value of the occurrence of external perturbations as proxies for manipulative experiments. The emergence and subsequent eradication of rinderpest resulted in multivariate transient dynamics, the pattern of which provides valuable information about the causal links that drive the system. At large spatial scales, manipulative experiments are infeasible, and deriving insights from natural experiments is an essential alternative for understanding the dynamics of complex systems at the landscape scale, which is a necessary step towards devising scientifically informed conservation policy in protected areas.

Our results also show that wildlife conservation (via control of illegal hunting and exotic diseases) has an evident potential to make the Serengeti a substantial C sink in both wood and soils. This status could possibly allow the Serengeti to draw revenue for its management; the annual amount of C removed from the atmosphere by the system operates as a sink that could offset seats taken by tourists on flights from Europe and the rest of the world to East Africa or be marketed as CO_2_ offsets on carbon markets. This suggests a novel approach to maintaining the conservation status of this region by coupling park revenues to the economics of C offsets. Furthermore, even though the current status of the Serengeti as a C sink is unlikely to hold indefinitely (the system will eventually saturate and become C-neutral), incentives are required that minimize the risk of the system becoming a net source of C should further disease outbreaks occur. The key point here is that the Serengeti may only work as an efficient C sink in the short term if it is grazed by over one million wildebeest (Figure 5C). Their abundance is intimately dependent upon the control of infectious diseases and game-meat poachers [36], as well as the continued viability of the migration, which is increasingly disrupted by land-use changes along the northern and western boundaries of the park [37]. The management of top-down trophic cascades can thus have important implications for how local ecological dynamics impact global-scale processes.

## Materials and Methods

### Study System and Data Sources

Serengeti National Park and the broader Serengeti-Mara ecosystem (Serengeti hereafter) have been described in detail elsewhere [11],[25],[38]. The ecosystem comprises an area of ∼25,000 km^2^ in Tanzania and Kenya in East Africa, and is characterized by a marked southeast to northwest rainfall gradient, as well as a roughly parallel gradient of increasing soil depth, sand to clay ratio, and declining fertility. It can be divided into areas of pure grassland in the southeastern plains and woodland in the rest of the ecosystem. The grasslands are the product of edaphic constraints [39], and the woodlands vary spatially and temporally in terms of tree cover [28],[40]. Wildebeest (*Connochaetes taurinus*) and elephants (*Loxodonta africana*) are dominant grazers and browsers, respectively, and can be regarded as keystone species in their respective feeding guilds, although giraffe (*Giraffa camelopardalis*) also have locally significant effects on trees [12].

We obtained wildebeest and elephant population estimates from census data [41],[42] for the entire Serengeti ecosystem, and calculated the proportion of area burned in any given year from published [28],[43] fire maps and our own database. We estimated mean per capita annual changes in tree density from sequential photopanoramas collected by A.R.E.S. at 51 sites in the Serengeti woodlands [11],[44] between 1960–2003 (Figure 1). The sites were chosen in northern and central Serengeti to match photopanoramas that had been established at earlier dates (pre-1960) and/or to achieve a good representation of road-accessible areas of the park. The time gaps between successive photopanoramas varied from site to site, and ranged between 2 and 31 y, resulting in sequences of between two and six photos per site (Dataset S1). To calculate observed annualized per capita changes in tree density (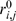) across sites (*i*) and time periods (*j*), we used the following equation:
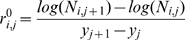
(1)where N_i,j_ and N_i,j+1_ are the numbers of trees counted within a fixed frame (reproducible across time periods) inserted in photos *j* and *j*+1, respectively, and *y*_j+1_−*y*_j_ is the time elapsed between photos. Note that N are counts, but because trees were counted within fixed areas, we could treat *r* as a density change. It is a relative and not absolute density change because we could not measure the absolute areas covered by the frames. We used monthly rain gauge data to generate rainfall surfaces with inverse distance weighting, and estimated mean ecosystem-wide annual rainfall (*R*_ann_, in mm), dry-season (June–October) rainfall (*R*_dry_, in mm), and the ratio of wet (November–May) to dry season rainfall (*R*_w∶d_) for the period 1960–2003. We used a poaching index (*P*, dimensionless) reconstructed from carcass data in the Serengeti [36] to model elephant population dynamics. We set *P* to 0 starting in 1990 on the basis of reports of negligible elephant poaching in the park following the ivory ban instituted in 1989. To incorporate the effects of atmospheric CO_2_ on tree population growth, we used published values of CO_2_ (*C*, in ppm) from the Mauna Loa long-term dataset in Hawaii [45]. We reconstructed the history of rinderpest seroprevalence in the Serengeti for the periods 1958–1963 and 1982–1989 from the literature [46]–[48]. Raw data values for the model covariates used in the analysis are given below as text files for R and WinBUGS input.

### State-Space Model

A technique that is increasingly gaining currency in ecological studies for the analysis of time series data with nonlinear dynamics, process and observation error, missing data, and latent variables is the BSS model using Gibbs sampling [49]–[52]. Given that our data analysis confronted all of these challenges, we adopted this approach to make inferences about the factors driving fire and tree population dynamics in the Serengeti. This framework allowed us to jointly model the population dynamics of the herbivores, which we treated as covariates, and fire and tree population dynamics. Some of the environmental covariates available for the Serengeti, such as annual rainfall, have been monitored continuously over the period of analysis, but herbivores have been censused unevenly over time; and for both elephants and wildebeest, the proportion of missing data exceeds 50%. To impute values for these missing data (with appropriate error estimates), we required nonlinear population dynamics models incorporating both process error (accounting for demographic and environmental uncertainty) and observation error [50].

There were four dynamic variables that needed to be modeled: the total numbers of wildebeest (*W*) and elephants (*E*), fire (*F*), expressed as the proportion of the ecosystem that burns year^−1^, and tree density ha^−1^ (T). The BSS model allowed us to model probability distributions for the true values of these variables, both for years with and without missing data, by specifying probability models for each variable in year *t* conditional on: (i) its value in year *t*−1; (ii) the values of other variables hypothesized to affect it; and (iii) the observations [50]. We treated *W* and *E* as modeled covariates and *F* and T as dependent variables. We modeled *W* and *E* by drawing on past work supporting key effects of dry-season rainfall on wildebeest carrying capacity [53], and of poaching [29] on elephant dynamics [54].

A BSS model generally comprises three components: a process equation describing the dynamics of the variable of interest (e.g., the true size of an animal population over time), an observation equation linking the process equation to the data, and prior distributions for the unknown parameters [50]. In this case, we have a multivariate time series of linked variables, so we have multiple process and observation equations [49]:

#### Process equations

Our model tracked the population dynamics of wildebeest, elephants, and trees, and the occurrence of fire. These variables can influence each others' dynamics (e.g., fire and elephants can affect trees), but each can also be influenced by a number of independent variables, which in our model included the various rainfall variables (*R*_ann_, *R*_dry_, and *R*_w∶d_), human hunting pressure (*P*), and atmospheric CO_2_ (*C*). We constructed our model on a foundation of extensive past research on the wildebeest of the Serengeti, which has shown that their population dynamics over the past half-century can be largely explained by release from rinderpest, followed by food limitation (grass production determined by dry season rainfall) rather than by predation or hunting, which have had a marginal effect [41],[53],[54],[55]. Rather than draw inferences on the regulation of herbivore populations, we are interested in reconstructing the trajectories (with error estimates) of these populations since 1960.

We used the logistic growth model for the deterministic portion of the wildebeest process equation:
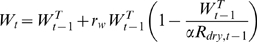
(2)Here, *W*_t_ and 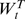 are the deterministic and true wildebeest population sizes at time *t*, respectively, and 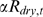 determines the carrying capacity of the system. We note that other models are possible, but Equation 2 (Figure 2A) fits the census data exceedingly well.

The Serengeti elephant population followed a pattern of rapid growth in the 1950s and 1960s, a decline due to poaching in the 1970s and 1980s, and subsequent recovery following the ivory ban in 1989. Elephant populations at other sites in Africa have exhibited consistently high population growth rates at population densities over an order of magnitude higher than those encountered in the Serengeti [56], so we assumed no density dependence. We used a simple model of exponential growth (adequate for the time period involved) coupled with a hunting term for the deterministic portion of the elephant equation:
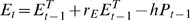
(3)where *E*_t_ and 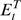 are the deterministic and true elephant population sizes at time *t*, respectively, *h* is a harvest parameter, and *P* is poaching intensity.

We use alternative forms of the following equation (depending on our candidate model) to model the proportion of the park that burns each year:
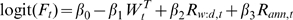
(4)Here, the logit link function keeps *F*_t_ within the bounds 0 (no fire) and 1 (complete burn). The term for 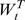 in Equation 4 assumes that wildebeest consumption affects the amount of grass biomass available for burning, and the term for *R*_w∶d_, on the basis of the premise that abundant wet season rain results in elevated fuel loads that are then more likely to burn under dry conditions in the dry season, follows from a hypothesized relationship between seasonal differences in rainfall distribution and fire [27],[57]. We also tested the effect of *R*_ann,t_ on fire, given that total grass production is primarily a function of annual rainfall [25],[58].

To model changes in tree density, we again use alternative formulations, with the “full” model being of the form:
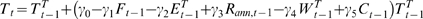
(5)where T*_t_* and 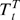 are the deterministic and true tree densities at time *t*, respectively. In each of the candidate models, one or more terms were dropped from Equations 4 and/or 5. The term containing 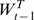 tested for a direct effect (in addition to the fire-mediated indirect effect) of wildebeest on tree dynamics, e.g., through trampling, consumption of seedlings, and damage through horning [14],[59], and *R_ann_* tested for the effect of wet years on recruitment pulses [60]. The variable C was included because CO_2_ concentration has increased significantly over the period of study [45], and it could contribute to CO_2_ fertilization and enhanced tree growth [4].

In Equations 2–5, the β's, γ's, α, *r*_W_, *r*_E_, and *h* are parameters to be estimated, together with *W*_0_, *E*_0_, and T_0_, the initial wildebeest and elephant population sizes and initial tree density, respectively. These equations represent a deterministic process. To introduce process error in the wildebeest, elephant, and tree population dynamics equations, we assumed lognormal errors (because of the geometric nature of population growth in Equations 2, 3, and 5) to derive the “true” population sizes at time *t*:
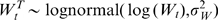
(6a)

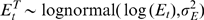
(6b)

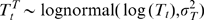
(6c)In time-series population data, ignoring process error can result in biases in parameter estimation because errors propagate through time [50]. We assume that population growth is a Markov process where the state of the population is conditionally dependent on its state in the preceding time period. Although process error is also bound to occur in the fire equation (fire occurrence is a stochastic process), ignoring process error poses less of a problem because we reasonably assume that *F*_t_ is independent of its value at *t*−1.

#### Observation equations

The parameters and process equations represent the unobserved portion of the model. Their values can be inferred by linking them to the data [50]. We assumed lognormal errors for the distributions of *W*, *E*, and *T* [49],[50]:
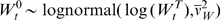
(7a)

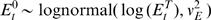
(7b)

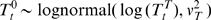
(7c)where 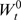 and 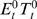 are wildebeest and elephant population estimates from census data and 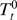 are observed tree densities. In addition to the population estimates, we have error estimates 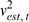 for the size of the wildebeest population for most census periods (Table 2). These can be used to inform the estimate of the true observation error for the size of the wildebeest population 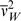. Clark and Bjornstad [50] used such estimates to generate priors for the size of the observation error for each period. We treated them as data, assuming that they represent alternative realizations from a single distribution of observation error with mean 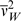. Given that variances are necessarily positive, we assumed an inverse gamma distribution for the estimated variances of the wildebeest census estimates:
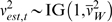
(8)The parameterization of the inverse gamma distribution in Equation 8 assumes a variance equal to the square of the mean [50].

Since our tree data consisted almost exclusively of per capita density changes, and not actual densities, we lacked data for 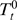. To provide an empirical reference point for tree density, we used data from a 1999 survey conducted across 113 plots in the woodland portion of the Serengeti [40]. This gave us a mean value of 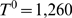 tree ha^−1^ in 1999. We used the standard error of tree density across these plots as a fixed value for 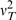.

We used a beta distribution to model error in the observation equation for fire because this variable (a proportion) is constrained to range between 0 and 1:
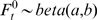
(9)We reparameterized Equation 9 in terms of the mean of the distribution (*F*_t_) as:
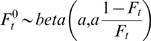
(10)leaving only parameter *a* to be estimated.

To model observation error in per capita tree density change, T^T^ was sampled by the model at the intervals *j* given by the photopanorama data (Equation 1) to estimate true values of 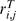:
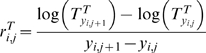
(11)These were then compared with the observed values (see Protocol S1):
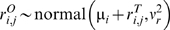
(12)where μ_i_ is a random effect that accounts for correlations among tree changes within site *i* (for example due to differences in fertility or topography among sites). We modeled μ_i_ as follows:
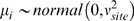
(13)The highly spatially clustered nature of the photopanorama dataset suggested that a random effect might be required to account for correlations among sites that are close together. To allow for this, we tested a model with a random effect for a “region effect” in *r* (north versus central Serengeti, Figure 1). The model was unable to converge on a solution for this coefficient (an identifiability issue [61]), suggesting either that the regional effect was negligible or that the dataset was too small to allow for an analysis of spatial effects. In our final analysis, we ignored such spatial correlations.

#### Priors

We used uninformative priors in all cases (see WinBUGS code below). We used uniform priors constrained by reasonable bounds (e.g., our initial population priors bracketed recent, pre-1960 census estimates) for the elephant and wildebeest population model parameters (α, *r*_W_, *r*_E_, *h*, *W*_0_, and *E*_0_) and for T_0_, Gaussian priors (with mean 0 and variance 10^6^) for the β's and γ's in the fire and tree equations (Equations 3 and 4), and inverse gamma distributions (with shape and scale = 10^−3^) for the variances of the process (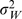, 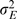, and 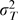) and observation (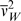, 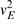, 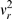, and 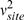) errors, and for parameter *a* of the beta distribution.

### Candidate Models and Implementation

Our immediate objective was to find 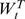, 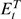, *F_t_*, and 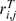 (the actual values of interest, which we can express as a vector ***X***) together with the model parameters and error estimates (which we jointly refer to as the vector **θ**) that produced the best fit to the data 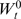, 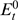, 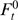, 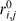 (the vector of observations ***Y***). The model described in Equations 2–13 can be expressed in terms of the joint likelihood of the variables and parameters for the period 1960–2003, given the observations, or *p*(***X***,***Y***| **θ**). The joint posterior distribution is proportional to this likelihood times the priors, and estimates of the ***X***'s and **θ**'s can be obtained by sampling from this joint posterior [50], which is difficult or impossible to do analytically. We used WinBUGS 1.4 [49],[62], which uses Gibbs sampling, a Markov Chain Monte Carlo (MCMC) technique [63], to generate these estimates. We ran ten versions of the model (Table 1), combining alternative forms of Equations 4 and 5, allowing for two different drivers of fire (wildebeest and wet∶dry rainfall ratio) and five of per capita tree density change (fire, elephants, rainfall, wildebeest impact not explained by effects on fire, and atmospheric CO_2_). We did not assess the potential contribution of human population increase on fire patterns for two reasons: first, we lacked sufficient data on human population change over the period in question; second, what we did have suggested that fire declined as the human population increased, making this explanation a poor a priori candidate for our fire model. We compared the fits of alternative models with the DIC, analogous to the AIC used in an information theoretic framework [64],[65]. Our alternative versions of Equations 4 and 5 allowed us to simultaneously determine the relative importance of climate and herbivory on fire occurrence, and of climate, herbivory, fire, and atmospheric CO_2_ on tree population dynamics. The WinBUGS code for the best model (model 3; see Table 1) is given in Protocol S1. We ran each model for 10^6^ iterations and discarded the first half of these as “burn-in.” We used multiple initial values for each parameter and checked for model convergence with the Gelman-Rubin diagnostic [66]. We verified that our sampling interval did not lead to autocorrelation between successive realizations of each variable. We also examined the posterior distributions of all model parameters and variables to ensure that that they were not unduly constrained by the limits imposed by the priors (in the case of uniform distributions) and that they were approximately normally distributed.

### Variance Explained and Effects of Density-Dependence

To put our results into perspective for readers unfamiliar with Bayesian approaches, we plotted observed versus predicted (by the state-space model) values for fire and tree cover change and calculated adjusted-*R* ^2^ values as approximate indicators of the amount of variance explained by the best model (Figures 1F and 1G). We took as our predicted values the mean of the posterior distribution for each response variable. Although the Bayesian approach generates distributions rather than point estimates, we treated these means as our best estimates of model predictions. We noted a number of outliers in the plot of observed versus predicted values of *r*_i,j_ (Figure 1G), even after accounting for site differences in tree population change. We hypothesized that these particularly high observed values of annualized relative growth might be associated with the initial tree densities in these sites, so we plotted the model residuals (*r*_obs_−*r*_pred_) against the logarithm of *N*_1_, the tree count at the beginning of each paired photo sequence. Although we found that initial tree abundance explained almost an additional fifth of the total variance in tree population growth (Figure 1H), we could not parameterize the exact magnitude of this effect because we were unable to standardize tree densities across photos.

### Estimation of Ecosystem C Flux

To estimate ecosystem-level C fluxes in the Serengeti as a result of changes in wildebeest population size, fire, and tree density, we simulated changes in the size of the two dominant ecosystem C pools, tree C, and SOC. Our own analysis indicated large shifts in tree density, and recent empirical and modeling studies support the existence of dominant fire and grazing effects on SOC (unpublished data) [18],[19], so we focused our analysis on these three effects. We explicitly simulated the dynamics of tree C to calculate annual changes in biomass C, and estimated gains/losses from the soil C pool caused by grazing and fire from equations derived empirically (unpublished data) and through modeling of soil nutrient dynamics [18], respectively. We did not explicitly model the dynamics of the SOC compartment because the fluxes we report are small in relation to the absolute size of the total soil C pool, and we could significantly simplify our analysis by treating SOC as a pool of constant size (to a first approximation) over the relatively short time scale of the analysis.

We modified the best overall state-space model (model 3 in Table 1 of the main text) by expressing tree density T in C units (Mg C km^−2^). We obtained a point estimate of tree C for 1999 (of 997 Mg C km^−2^) in the woodland portion of the ecosystem by combining our plot data [40] with allometric equations relating stem and crown diameter with aboveground and belowground biomass in *Acacia tortilis* [67], the most common tree species in the ecosystem. We then converted biomass into tree C per km^2^ in the survey plots across tree size classes. Fire effects vary widely across tree size classes [12], and much of the woody biomass in large trees does not burn and volatilize in the short term [68]. Because our tree data does not discriminate among size classes, however, we can not incorporate this size distribution effect and treat our estimates of biomass C fluxes only as approximations. We used the estimated 1999 value in combination with the model to estimate woody biomass C for the entire period 1960–2003, the same way we previously did with density.

To simulate the effect of grazing on the soil C pool, we used the following empirically derived polynomial equation (unpublished data) relating SOC flux to (GI):
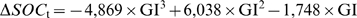
(14)where ΔSOC is in units of Mg C km^−2^ y^−1^ and GI equals the proportion of aboveground net primary production (N*PP*_t_, grasses only) consumed by grazers (*CO*N*S*_t_). To estimate GI we first had to estimate N*PP*_t_ and *CO*N*S*_t_ on the basis of rainfall and the size of the wildebeest population. We used an empirically derived equation relating N*PP*_t_ to annual rainfall (*R*_ann_) to estimate annual production [25] in Mg km^−2^ y^−1^:
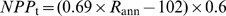
(15)The correction factor of 0.6 adjusts the production estimate to account for bare ground, topography, rivers, etc. [21],[38]. To estimate *CO*N*S*_t_ (in MG DM km^−2^ y^−1^) on a unit area basis (assuming a total area of 25,000 km^2^) we used the following equation:
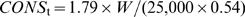
(16)where 1.79 (in Mg DM) is our estimate of annual consumption for an average wildebeest based on empirically derived functions relating daily voluntary intake to body mass [69]. In our analysis we only model wildebeest, but numerous other grazing species (such as buffalo) have covaried numerically with wildebeest as a result of rinderpest eradication and poaching pressure [15],[36]. On the basis of census data, we estimate that wildebeest represent 54% of the biomass of Serengeti grazers on a metabolic basis (which maps to consumption), and use this value in Equation 16 to generate a realistic estimate of historic consumption patterns for all grazers. We used Equations 15 and 16 to estimate GI = *CO*N*S*_t_/N*PP*_t_ on an annual basis, and applied this estimate to Equation 14 to estimate Δ*SOC*_t_. Our mean simulated estimate of GI for the period 1974–1977 (0.55) compared favorably with a mean field-based estimate of 0.52 obtained for this period [25].

To estimate Δ*SOC*_t_ as a function of fire, we first used a published model of Serengeti soil organic matter (SOM) dynamics [18] to estimate mean annual SOM changes in the top 10 cm of soil (the layer most susceptible to fire-induced SOM losses [70]) as a function of fire frequency. We estimated maximum annual SOM (and SOC) losses of 0.8% y^−1^ with an annual fire regime (*F*_t_ = 1). These estimates are consistent with long-term values measured elsewhere [71]. We used a linear interpolation (with Δ*SOC*_t_ = 0 with no fire) to estimate Δ*SOC*_t_ as a function of area burned (*F*_t_), as follows:
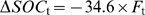
(17)based on mean values of SOM of 7.8% [19] and a mean bulk density of 1.21 [25].

To estimate changes in total ecosystem C, we modified our best-fit state-space model (model 3 in Table 1) to simulate Δ tree *C*_t_+Δ*SOC*_t_ over the period 1960 to 2003 based on inferred values of *W*_t_, *F*_t_, and T_t_ and Equations 14–17. We adjusted Δ tree *C*_t_ in our calculations of total ecosystem C change by a factor of 2/3 to account for the fact that one third of the ecosystem consists of edaphic, tree-less grasslands. To smooth out the high degree of inter-annual variation in Δ tree *C*_t_+Δ*SOC*_t_, we present our results as mean annual changes calculated over decadal intervals.

## Supporting Information

Dataset S1 **Time-series data for model variables used in the analysis.**
(0.04 MB DOC)

Protocol S1 **R and WinBUGS computer code used for the BSS model.**
(0.06 MB DOC)

## Abbreviations

BSS: Bayesian state-space model
DIC: deviance information criterion
GI: grazing intensity
SOC: soil organic carbon
SOM: soil organic matter
