# Supplementary material for: A Disease-Mediated Trophic Cascade in the Serengeti and its Implications for Ecosystem C
Source: PLoS Biol. 2009 Sep 29;7(9):e1000210. doi: 10.1371/journal.pbio.1000210 (PMC2740867; doi:10.1371/journal.pbio.1000210)
Supplement: Dataset S1 — Time-series data for model variables used in the analysis. (0.04 MB DOC) [file pbio.1000210.s001.doc]

**Dataset S1**

The following data file contains the time series data for rainfall (*R_ann_* = annual rainfall, *R_dry_* = dry-season rainfall, *R_wet_* = wet-season rainfall, all in mm y^-1^), elephant population size *E* (and predicted elephant population *E_pred_* for initializing the model), wildebeest population size (*Wobs*, in thousands, and predicted wildebeest population *W_pred_*), the estimated standard error of *W* (expressed as a tau = 1/var), fire *F* (as the proportion of Serengeti that burns each year) between 1960 and 2003, tree density (Tobs, in trees ha^-1^, and predicted tree density Tpred), and atmospheric CO_2_ (in ppm).

**Model_data1.txt**

Rann Rdry Rwd Eobs Epred P Wobs Wpred tauest Fobs Tobs Tpred CO2

1960 920 129 6.14 NA 1320 0.02 NA 286 NA NA NA 366.5 316.91

1961 829 110 6.57 1155 1405 0.02 267 325 NA NA NA 337.6 317.65

1962 1308 251 4.21 NA 1495 0.02 NA 373 NA NA NA 309.5 318.45

1963 831 64 11.90 NA 1591 0.02 357 420 NA 0.71 NA 299.2 318.99

1964 912 152 5.02 NA 1692 0.02 NA 472 NA 0.46 NA 250.5 319.52

1965 656 159 3.11 2071 1801 0.02 439 518 NA NA NA 240.2 320.03

1966 938 137 5.84 2216 1916 0.02 NA 577 NA 0.86 NA 240 321.37

1967 700 96 6.27 2474 2039 0.02 484 626 NA 0.70 NA 226.6 322.18

1968 1090 121 8.02 2278 2169 0.02 NA 697 NA 0.72 NA 211.3 323.05

1969 605 92 5.60 NA 2308 0.02 NA 731 NA 0.53 NA 195.4 324.62

1970 912 122 6.48 3189 2455 0.02 NA 796 NA 0.67 NA 189 325.68

1971 734 168 3.36 NA 2613 0.02 693 842 578.1 0.36 NA 179.8 326.32

1972 870 312 1.79 2800 2780 0.02 773 901 102.1 0.40 NA 182.9 327.46

1973 924 201 3.61 3000 2958 0.02 NA 964 NA 0.50 NA 185 329.68

1974 916 220 3.16 3320 3147 0.02 NA 1024 NA 0.15 NA 189.5 330.25

1975 776 224 2.46 2328 3349 0.02 NA 1057 NA 0.06 NA 198.6 331.15

1976 775 207 2.74 NA 3563 0.02 NA 1087 NA 0.12 NA 207.1 332.15

1977 847 164 4.18 2500 3584 0.06 1443 1127 52.3 0.27 NA 213.8 333.9

1978 908 173 4.26 NA 3294 0.12 1251 1174 12.9 NA NA 222.4 335.5

1979 883 106 7.33 NA 2931 0.14 NA 1212 NA NA NA 229.4 336.85

1980 782 122 5.39 NA 2481 0.17 1337 1222 280.2 NA NA 231.9 338.69

1981 825 121 5.81 NA 2064 0.18 NA 1241 NA NA NA 249.1 339.93

1982 676 161 3.21 NA 1687 0.19 1212 1212 20.3 0.05 NA 265.9 341.13

1983 803 201 2.99 NA 1379 0.19 NA 1227 NA NA NA 295 342.78

1984 641 152 3.22 1834 1148 0.18 1337 1188 94.3 NA NA 327.3 344.42

1985 776 129 5.00 NA 971 0.17 NA 1200 NA 0.23 NA 359.9 345.9

1986 724 118 5.14 467 850 0.15 1148 1195 73.8 0.31 NA 395 347.15

1987 912 139 5.56 NA 769 0.13 NA 1236 NA 0.30 NA 415.3 348.93

1988 894 161 4.57 NA 707 0.12 NA 1269 NA 0.23 NA 485.9 351.48

1989 861 185 3.65 500 680 0.09 NA 1290 92.6 NA NA 591.3 352.91

1990 913 135 5.78 NA 744 0 NA 1320 NA NA NA 667.3 354.19

1991 735 188 2.90 NA 813 0 1225 1296 48.0 NA NA 735 355.59

1992 680 163 3.16 980 888 0 NA 1256 NA NA NA 763.7 356.37

1993 679 63 9.86 NA 971 0 NA 1225 NA NA NA 835.5 357.04

1994 682 157 3.34 1357 1061 0 919 1201 28.4 NA NA 825.7 358.88

1995 777 165 3.72 NA 1160 0 NA 1211 NA NA NA 876.9 360.88

1996 838 239 2.51 NA 1268 0 NA 1235 NA NA NA 931 362.64

1997 551 77 6.16 NA 1386 0 NA 1149 NA NA NA 1002 363.76

1998 1004 143 6.03 2015 1515 0 924 1209 22.0 NA NA 1025 366.63

1999 619 96 5.45 NA 1656 0 1298 1165 NA 0.50 1260 1044 368.31

2000 475 81 4.86 1613 1810 0 1249 1055 74.3 0.10 NA 1135 369.48

2001 838 191 3.40 NA 1978 0 NA 1097 NA 0.27 NA 1190 371.02

2002 759 79 8.63 NA 2162 0 NA 1118 NA 0.49 NA 1235 373.1

2003 896 168 4.33 2360 2363 0 NA 1164 NA 0.29 NA 1221 375.64

The following data file contains the tree density photopanorama data collected by A.R.E.S. between 1960-2003: ph represents photos for a particular site, and int represents a time interval for that site, spanning years y1 and y2, where 1960 = 1; *R_obs_* is the observed change in tree density, on a log scale (see eq. 1).

**Model_data2.txt**

Data points reserved for model validation in the second analysis (see Fig. 3 of the main text) are shown in bold.

ph int Robs y1 y2

1 1 1 0.070 21 32

2 1 2 0.089 32 44

3 2 1 0.055 21 32

4 2 2 0.016 32 44

5 3 1 0.047 32 44

6 4 1 0.037 21 27

7 4 2 0.030 27 32

8 4 3 0.015 32 44

9 5 1 0.054 21 27

10 5 2 0.028 27 32

11 5 3 0.022 32 44

35 13 1 0.019 21 32

36 13 2 0.037 32 39

37 13 3 0.079 39 41

38 14 1 0.042 21 32

39 14 2 0.036 32 39

40 14 3 0.000 39 41

**41 15 1 -0.029 8 21**

42 15 2 0.016 21 25

43 15 3 0.049 25 32

44 15 4 0.040 32 39

**45 16 1 -0.066 8 23**

46 16 2 0.118 23 25

47 16 3 0.087 25 32

48 16 4 0.055 32 39

55 20 1 0.130 21 41

56 21 1 0.078 21 41

57 22 1 0.265 21 41

58 23 1 0.060 21 41

59 24 1 0.077 21 41

60 25 1 0.093 21 41

61 26 1 0.071 32 44

**62 27 1 0.055 13 39**

75 32 1 0.120 23 32

76 32 2 0.044 32 40

77 33 1 0.064 27 41

78 34 1 0.018 21 27

79 34 2 0.089 27 32

80 34 3 0.048 32 44

81 35 1 0.113 21 27

82 35 2 0.303 27 32

83 35 3 0.016 32 44

84 36 1 0.064 21 27

85 36 2 0.156 27 32

86 36 3 0.008 32 44

**87 37 1 0.150 6 21**

88 37 2 -0.025 21 27

89 37 3 0.135 27 33

90 37 4 0.026 33 44

**91 38 1 -0.093 6 21**

92 38 2 0.213 21 27

93 38 3 0.067 27 33

94 38 4 0.075 33 44

**95 39 1 -0.077 6 21**

96 39 2 0.226 21 27

97 39 3 0.057 27 33

98 39 4 0.060 33 44

**99 40 1 0.008 6 21**

100 40 2 0.153 21 27

101 40 3 0.044 27 33

102 40 4 0.068 33 44

103 41 1 0.051 21 27

104 41 2 0.089 27 32

105 41 3 0.047 32 44

106 42 1 0.165 21 27

107 42 2 0.112 27 32

108 42 3 0.028 32 44

109 43 1 0.440 21 27

110 43 2 0.288 27 32

111 43 3 0.021 32 44

**114 46 1 0.061 13 44**

**115 47 1 0.050 13 44**

**116 48 1 0.038 13 44**

**117 49 1 -0.035 8 18**

**118 49 2 0.023 18 29**

119 49 3 0.137 29 32

120 49 4 0.019 32 41

121 50 1 0.053 27 41

122 51 1 0.169 27 41

12 6 1 0.025 21 29

13 6 2 0.066 29 44

14 7 1 0.084 21 28

15 7 2 0.189 28 32

16 7 3 0.052 32 44

17 8 1 0.093 21 28

18 8 2 0.446 28 32

19 8 3 0.032 32 39

20 9 1 0.058 21 28

21 9 2 0.052 28 32

22 9 3 0.012 32 40

23 9 4 0.023 40 44

24 10 1 0.007 32 44

25 11 1 0.041 21 27

26 11 2 0.066 27 30

27 11 3 0.037 30 32

28 11 4 0.087 32 40

29 11 5 0.025 40 44

30 12 1 0.075 21 27

31 12 2 0.203 27 30

32 12 3 0.064 30 32

33 12 4 0.014 32 40

34 12 5 0.021 40 44

49 17 1 0.116 27 32

50 17 2 0.050 32 44

51 18 1 0.072 27 32

52 18 2 0.041 32 44

53 19 1 0.542 27 32

54 19 2 0.176 32 44

**63 28 1 -0.005 9 19**

**64 28 2 0.041 19 29**

65 28 3 0.023 29 40

**66 29 1 0.002 9 19**

**67 29 2 0.061 19 30**

68 29 3 0.052 30 40

**69 30 1 -0.024 9 19**

**70 30 2 0.081 19 30**

71 30 3 0.034 30 40

**72 31 1 -0.007 9 19**

**73 31 2 0.066 19 30**

74 31 3 0.026 30 40

**112 44 1 -0.092 1 9**

**113 45 1 -0.126 1 9**
