# Supplementary material for: A Disease-Mediated Trophic Cascade in the Serengeti and its Implications for Ecosystem C
Source: PLoS Biol. 2009 Sep 29;7(9):e1000210. doi: 10.1371/journal.pbio.1000210 (PMC2740867; doi:10.1371/journal.pbio.1000210)
Supplement: Protocol S1 — R and WinBUGS computer code used for the BSS model. (0.06 MB DOC) [file pbio.1000210.s002.doc]

# Protocol S1

R code used to run WinBUGS through the R2WinBUGS package. We show the R script and WinBUGS code for model 3 (Table 1 of the main text).

**R script**

library (R2WinBUGS)

library (emdbook)

# input rainfall, animal, and fire data

ssdat1 <- read.table("Model_data1.txt")

Rann <- ssdat1$Rann # annual rainfall in mm

Rdry <- ssdat1$Rdry # dry season rainfall in mm

Rwd <- ssdat1$Rwd # ratio of wet:dry season rainfall

E.obs <- ssdat1$Eobs # elephant population size

Epred <- ssdat1$Epred # deterministic estimates of elephant population size used as initial conditions

Pe <- ssdat1$P # poaching index

W.obs <- ssdat1$Wobs # wildebeest in thousands

Wpred <- ssdat1$Wpred # deterministic estimates of wildebeest population size used as initial conditions

tauW.est <- ssdat1$tauest # observation error estimate for wildebeest (expressed as a lognormal precision=1/variance)

F.obs <- ssdat1$Fobs # proportion of ecosystem burned

T.obs <- ssdat1$Tobs # tree density (ha^-1^)

Tpred <- ssdat1$Tpred # deterministic estimates of tree density used as initial conditions

Atm.CO2 <- ssdat1$CO2 # atmospheric CO_2_ in ppm

N1 = length(W.obs)

# input photopanorama data and arrange in an array (rows=photo site, columns=time interval)

ssdat2 <- read.table("Model_data2.txt")

photo <- ssdat2$ph

interv <- ssdat2$int

robs <- ssdat2$Robs

year1 <- ssdat2$y1

year2 <- ssdat2$y2

N2 <- max(photo)

N3 <- rep(0,N2)

R.obs <- rep(NA,255)

dim(R.obs) <- c(51,5)

Y1 <- rep(NA,255)

dim(Y1) <- c(51,5)

Y2 <- rep(NA,255)

dim(Y2) <- c(51,5)

for (k in 1:255){

i <- photo[k]

j <- interv[k]

N3[i] = N3[i]+1

R.obs[i,j] <- robs[k]

Y1[i,j] <- year1[k]

Y2[i,j] <- year2[k]

}

# model 3

# variables sent to WinBUGS (only those used in model 3 are sent)

tree.data <- list("N1", "Rdry", "Rwd", "W.obs", "F.obs", "E.obs", "Pe", "tauW.est", "N2", "N3", "R.obs", "Y1", "Y2", “T.obs”)

# initial conditions

inits <- list(list(T0 = 500, T.true = Tpred, W0 = 245, rw = 0.12, alpha = 9.0, beta1= -0.0022, beta0 = 1.4, beta2 = 0.023, gamma0 = 0.25, gamma1 = 0.45, tauW.obs.mean = 115, tauW.proc = 120, tauR.obs = 200, tau.site = 900, tauT.proc = 170, a1 = 2.2, W.true = Wpred, E0 = 1025, re = 0.09, he = 900, tauE.proc = 30, tauE.obs = 200, E.true = Epred),

list(T0 = 400, T.true = Tpred+rnorm(N1,0,10), W0 = 235, rw = 0.16, alpha = 9.2, beta1= -0.0025, beta0 = 1.32, beta2 = 0.025, gamma0 = 0.17, gamma1 = 0.5, tauW.obs.mean = 120, tauW.proc = 550, tauR.obs = 230, tauT.proc = 180, tau.site = 820, a1 = 2.0, W.true = Wpred + rnorm(N1,0,25), E0 = 1050, re = 0.07, he = 1100, tauE.proc = 20, tauE.obs = 220, E.true = Epred+rnorm(N1,0,10)),

list(T0 = 600, T.true = Tpred+rnorm(N1,0,10), W0 = 240, rw = 0.18, alpha = 8.5, beta1= -0.0027, beta0 = 1.3, beta2 = 0.02, gamma0 = 0.2, gamma1 = 0.47, tauW.obs.mean = 110, tauW.proc = 650, tauR.obs = 180, tauT.proc = 200, tau.site = 850, a1 = 2.5, W.true = Wpred + rnorm(N1,0,25), E0 = 1000, re = 0.08, he = 1000, tauE.proc = 25, tauE.obs = 180, E.true = Epred+rnorm(N1,0,10)))

# WinBUGS output desired

parameters <- c("E.true", "W.true", "F.det", "T.true", "mu.site", "rtrue", "T0", "W0", "rw", "alpha", "tauW.obs.mean", "nuW", "tauW.proc", "sigmaW", "a1", "beta0", "beta1", "beta2", "gamma0", "gamma1", "tau.site", "nuSITE", "tauR.obs", "nuR", "tauT.proc", "sigmaT ", "E0", "re", "he", "sigmaE", "tauE.proc", "nuE", "tauE.obs")

# send model, initial conditions, and data to WinBUGS

ssmod <- bugs(data=tree.data,inits,param=parameters, model="model3.txt",n.chains=length(inits),n.iter=300000,n.burnin=150000)

# test for convergence

ssdiag = as.mcmc.bugs(ssmod)

print(gelman.diag(ssdiag,transform=TRUE))

# print output

print(treesim,digits=6)

**WinBUGS code**

WinBUGS code for Bayesian state-space model (model 3 from Table 1 in the main text is shown here). Note that variances in normal and lognormal distributions are expressed as taus (inverse variances). The variable tauW.est represents the standard error estimates from the wildebeest population censuses transformed into inverse variances in a lognormal model, for example. N1, N2, and N3 represent the number of years with data (N1 = 44, from 1960 to 2003), the number of photopanorama sites (N2 = 51), and the number of photo sequences per site (N3 = 1-5). In our analysis, we treated wildebeest and elephants as covariates and assumed causal effects on fire and trees, the dependent variables. We formalized this in WinBUGS by applying the “cut” function to $W_{t}^{T}$and $E_{t}^{T}$to obtain $W_{t}^{cut}$and$E_{t}^{cut}$, which we substituted for $W_{t}^{T}$and $E_{t}^{T}$in Eqs. 3 and 4 [1]. This ensured that estimates of *W* and *E* were estimated only from census, rainfall, and poaching data, and not constrained by *F* and *r* [2]. We also constrained some of the equations above with “max” functions to keep populations from acquiring nonsensical negative values.

## model 3

model {

## initial values

W.det[1] <- W0

logWdet[1] <- log(W.det[1])

W.true[1] ~ dlnorm(logWdet[1],tauW.proc)

E.det[1] <- E0

logEdet[1] <- log(E.det[1])

E.true[1] ~ dlnorm(logEdet[1],tauE.proc)

T.det[1] <- T0

logTdet[1] <- log(T.det[1])

T.true[1] ~ dlnorm(logTdet[1],tauT.proc)

## process model

for (i in 2:N1){

W.cut[i-1] <- cut(W.true[i-1])

E.cut[i-1] <- cut(E.true[i-1])

## elephants

E.det[i] <- max(E.true[i-1]+E.true[i-1]*re-he*Pe[i-1],1)

logEdet[i] <- log(E.det[i])

E.true[i] ~ dlnorm(logEdet[i],tauE.proc)

## wildebeest

K[i-1] <- Rdry[i-1]*alpha

W.det[i] <- max(W.true[i-1]+rw*W.true[i-1]*(1-W.true[i-1]/K[i-1]),1)

logWdet[i] <- log(W.det[i])

W.true[i] ~ dlnorm(logWdet[i],tauW.proc)

## fire

logit(F.det[i-1]) <- beta0 + W.cut[i-1]*beta1 + Rwd[i-1]*beta2

## trees

T.det[i] <- max(T.true[i-1] + T.true[i-1]*(gamma0 - F.det[i-1]*gamma1),1)

logTdet[i] <- log(T.det[i])

T.true[i] ~ dlnorm(logTdet[i],tauT.proc)

rtrue[i] <- log(T.true[i])-log(T.true[i-1])

}

W.cut[N1] <- cut(W.true[N1])

E.cut[N1] <- cut(E.true[N1])

logit(F.det[N1]) <- beta0 + W.cut[N1]*beta1 + Rwd[N1]*beta2

## elephant, wildebeest, fire, and tree data models

for (i in 1:N1){

logEtrue[i] <- log(E.true[i])

E.obs[i] ~ dlnorm(logEtrue[i],tauE.obs)

logWtrue[i] <- log(W.true[i])

W.obs[i] ~ dlnorm(logWtrue[i],tauW.obs.mean)

tauW.est[i] ~ dgamma(tauW.obs.shape,tauW.obs.rate)

F.det2[i] <- max(0.00001,min(0.99999,F.det[i]))

b[i] <- a*(1-F.det2[i])/F.det2[i]

F.obs[i] ~ dbeta(a,b[i])

logTtrue[i] <- log(T.true[i])

T.obs[i] ~ dlnorm(logTtrue[i],58) # 58 is the lognormal tau for the SE for tree density

}

## data model for per capita tree density changes over time

for (i in 1:N2){

for (j in 1:N3[i]){

logRtrue[i,j] <- (log(T.true[Y2[i,j]])-log(T.true[Y1[i,j]]))/(Y2[i,j]-Y1[i,j])

sitemean[i,j] <- mu.site[i]+logRtrue[i,j]

R.obs[i,j] ~ dnorm(sitemean[i,j],tauR.obs)

}

mu.site[i] ~ dnorm(0.0,tau.site)

}

## priors

E0 ~ dunif(750,1250)

re ~ dunif(0.01,2)

he ~ dunif(0,5000)

W0 ~ dunif(200,300)

rw ~ dunif(0.01,2)

alpha ~ dunif(0.01,50)

T0 ~ dunif(1,1000)

beta0 ~ dnorm(0.0,0.000001)

beta1 ~ dnorm(0.0,0.000001)

beta2 ~ dnorm(0.0,0.000001)

gamma0 ~ dnorm(0.0,0.000001)

gamma1 ~ dnorm(0.0,0.000001)

tauE.obs ~ dgamma(0.001,0.001)

tauE.proc ~ dgamma(0.001,0.001)

tauW.obs.mean ~ dgamma(0.001,0.001)

tauW.obs.shape <- 1

tauW.obs.rate <- tauW.obs.shape/tauW.obs.mean

tauW.proc ~ dgamma(0.001,0.001)

tauR.obs ~ dgamma(0.001,0.001)

tauT.proc ~ dgamma(0.001,0.001)

tau.site ~ dgamma(0.001,0.001)

a ~ dgamma(0.001,0.001) ## fire beta distribution parameter

sigmaW <- 1/sqrt(tauW.proc) ## wildebeest process error

sigmaE <- 1/sqrt(tauE.proc) ## elephant process error

sigmaT <- 1/sqrt(tauT.proc) ## tree process error

nuW <- 1/sqrt(tauW.obs.mean) ## mean wildebeest observation error

nuE <- 1/sqrt(tauE.obs) ## elephant observation error

nuR <- 1/sqrt(tauR.obs) ## tree observation error

nuSITE <- 1/sqrt(tau.site) ## site observation error

}

References

1. Spiegelhalter D, Thomas A, Best N, Lunn D (2003) WinBUGS User Manual.

2. Carrigan G, Barnett AG, Dobson AJ, Mishra G (2007) Compensating for missing data from longitudinal studies using WinBUGS. Journal of Statistical Software 19: 1-17.
